# Community context for mechanisms of disease dilution: insights from linking epidemiology and plant–soil feedback theory

**DOI:** 10.1111/nyas.14325

**Published:** 2020-03-13

**Authors:** Cathy D. Collins, James D. Bever, Michelle H. Hersh

**Affiliations:** ^1^ Biology Program Bard College Annandale‐on‐Hudson New York; ^2^ Department of Ecology and Evolutionary Biology University of Kansas Lawrence Kansas; ^3^ Kansas Biological Survey University of Kansas Lawrence Kansas; ^4^ Department of Biology Sarah Lawrence College Bronxville New York

**Keywords:** species coexistence, pathogen, dilution effect, disease ecology, feedbacks, trophic interactions

## Abstract

In many natural systems, diverse host communities can reduce disease risk, though less is known about the mechanisms driving this “dilution effect.” We relate feedback theory, which focuses on pathogen‐mediated coexistence, to mechanisms of dilution derived from epidemiological models, with the central goal of gaining insights into host–pathogen interactions in a community context. We first compare the origin, structure, and application of epidemiological and feedback models. We then explore the mechanisms of dilution, which are grounded in single‐pathogen, single‐host epidemiological models, from the perspective of feedback theory. We also draw on feedback theory to examine how coinfecting pathogens, and pathogens that vary along a host specialist–generalist continuum, apply to dilution theory. By identifying synergies among the feedback and epidemiological approaches, we reveal ways in which organisms occupying different trophic levels contribute to diversity–disease relationships. Additionally, using feedbacks to distinguish dilution in disease incidence from dilution in the net effect of disease on host fitness allows us to articulate conditions under which definitions of dilution may not align. After ascribing dilution mechanisms to macro‐ or microorganisms, we propose ways in which each contributes to diversity–disease and productivity–diversity relationships. Our analyses lead to predictions that can guide future research efforts.

## Graphical abstract

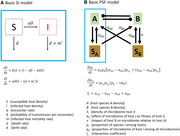

## Introduction

The quest to understand disease dynamics in ecological communities has gained attention in recent decades given the dramatic impact anthropogenic activities have on biodiversity, community composition, and species interactions.^1^, ^2^, ^3^, ^4^ Models that consider disease in a community context typically aim to (1) predict the impact of diversity on disease risk (how communities affect disease) or (2) predict the impact pathogens have on host diversity (how disease affects communities). While these goals are inherently connected because feedbacks exist between host community structure and pathogen dynamics,^5^ the two research trajectories have largely been advanced using different modeling approaches.

Efforts to understand the impact species diversity has on disease spread can be traced back centuries to agriculturalists aiming to minimize crop losses from disease and other pests.^6^ Experimental work later showed the value of mixing genetic lines^7^, ^8^ and crop species^9^ for minimizing disease. Ecologists observing a similar phenomenon in natural systems, that is, lower disease in communities with higher species diversity,^10^ employed epidemiological (e.g., SI/SIR) models to generate predictions and describe mechanisms through which diversity mediates disease spread (e.g., “dilution”^11^, ^12^). The suite of mechanisms leading to dilution was originally described for a disease system governed by a single, *host‐specific* *pathogen*,^11^ although dilution has been applied more broadly to interpret disease patterns in communities with multiple hosts that vary in *competence*.^1^, ^10^, ^13^

Appreciation for the influence disease exerts on species diversity emerged more recently, initially revealed through empirical and theoretical work showing that natural enemies contribute to species coexistence.^14^, ^15^, ^16^, ^17^, ^18^ Substantial evidence has accrued that pathogens play a key role in maintaining diversity in plant communities,^19^ and can play an important role in coexistence in animal communities as well.^20^, ^21^ Progress investigating the role of pathogens in structuring natural communities was accelerated with the development of feedback theory that describes plant–microbe interactions, often referred to as plant–soil feedback (PSF) theory.^22^, ^23^, ^24^, ^25^, ^26^ Models using this framework represent the host‐specific changes in plant/soil microbiome (including pathogens), and identify the conditions for microbiome‐mediated coexistence of host species. Host species coexistence is possible when the host‐specific effects of the microbiome negatively impact the fitness of conspecific neighbors more than heterospecific neighbors.^22^

Epidemiological models and feedback models represent many of the same phenomena, but their structure, terminology, and parameters reflect their distinct goals, as well as the disciplinary origin of each framework (epidemiology and community ecology, respectively). What insights can be gained by merging these perspectives? Our objective is to relate the feedback framework to dilution mechanisms grounded in epidemiological models. We first describe basic epidemiological and feedback frameworks, comparing and contrasting their construction and assumptions. Building on Keesing *et al*.,^11^ we then explore mechanisms through which diversifying communities may reduce *disease incidence* through the lens of feedback theory developed by Bever *et al*.^22^ We conclude by posing specific predictions that emerge from our analysis and suggest fruitful avenues for future research.

## Background: the disease ecology frameworks

### Disease dynamics: epidemiological approach

**Figure 1:**
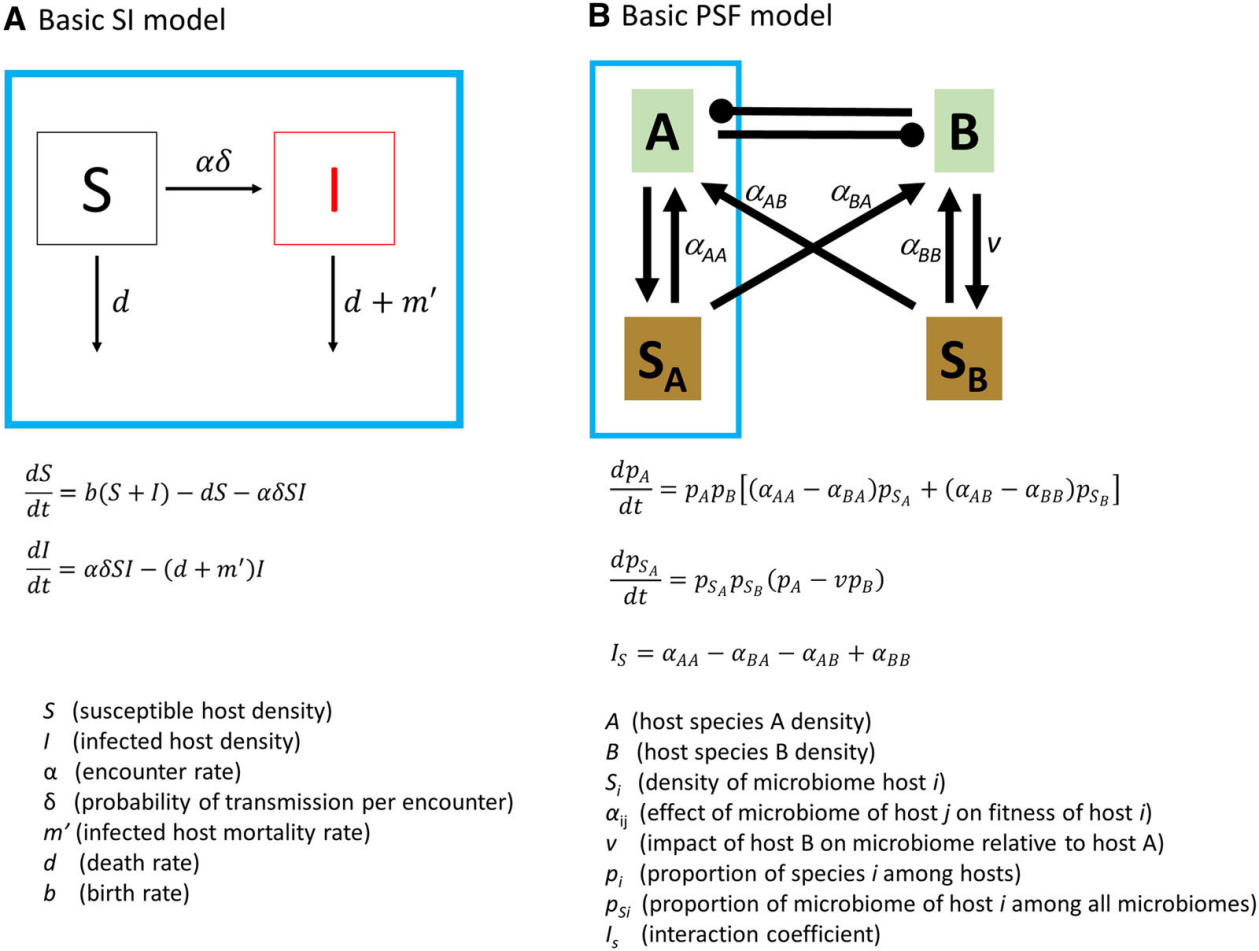
Conceptual diagram of SI (A) and feedback (B) modeling frameworks. In (A), boxes represent the states of individual hosts (susceptible and infected), while arrows show demographic changes or transitions between states (transmission). Arrows depicting birth rates are omitted for simplicity. In (B), green boxes represent host populations regardless of infection status, while brown boxes represent the entire microbiome of each host, including all pathogens, as well as other plant‐associated microbes regardless of function. Arrows depict the direction of beneficial effects, and clubs depict the direction of detrimental effects. In this example, all effects occur with similar magnitude; however, the thickness of the line can be altered to indicate the relative strength of effects. The blue boxes in each model highlight the area of conceptual overlap between the epidemiological and feedback frameworks. Under feedbacks, both boxes S and I from the epidemiological model are incorporated into green‐shaded box B (host population), while the arrow from A to S_A_ represents transmission (αδ in the epidemiological framework, often referred to as β). Conditions for host species coexistence in feedbacks are described by *I*_s_. It is important to note that the symbol *I* is used in both models, but holds different meanings in each. In the SI model, *I* represents the proportion of individuals infected, whereas in the feedback model, *I*_s_ is the interaction coefficient that reflects the net effect of the soil microbiome on hosts and competing species.

Efforts to model disease in a community context often build on population‐level epidemiological models constructed with the SI/SIR framework.^27^, ^28^ In these compartmental models, all individuals in a population are classified into states, such as *susceptible hosts*, *infected hosts*, or recovered hosts (when applicable) (Fig. 1A). As individuals transition between states over time, models quantify both key disease parameters (such as *transmission* and *recovery* rates) and disease‐induced changes to demographic rates, such as the amount of disease‐induced mortality. An important term often estimated is the basic reproductive number *R*_0_, which calculates how quickly an epidemic would spread in a fully susceptible population. The simplest models involve a single host, a single pathogen, and a limited number of states. However, models can be expanded to allow for the complexity of real‐world disease outbreaks.^29^, ^30^ This could include adding additional states for the population (e.g., exposed and quarantined), allowing for variable transmission rates among individuals, or incorporating vector‐borne or sexually transmitted diseases, which can generate *frequency‐dependent* rather than *density‐dependent* transmission.^31^ Some aspects of disease biology, such as *environmental* transmission,^32^, ^33^ prove more difficult to model. Still, this approach has proven to be enormously useful in understanding the dynamics of human, wildlife, and plant diseases, and the management of epidemics.^34^

Single‐pathogen epidemiological models can be expanded to the community level to include additional host species.^35^, ^36^, ^37^ When multihost pathogens are considered, hosts can vary in their susceptibility to the pathogen as well as their competence,^38^, ^39^ or their capacity to transmit infection. More complexity can be added by incorporating nonhost species (e.g., predators, competitors, mutualists, and resource species) that interact with a given host.^11^, ^38^, ^40^, ^41^, ^42^ These interactions between hosts and nonhosts can increase or decrease *disease prevalence*, through changes in the number of individuals in a given state (e.g., susceptible host regulation and infected host mortality) or changes in the transitions between states (e.g., encounter reduction/augmentation, transmission reduction, and recovery augmentation).^11^, ^42^ Additional species—both hosts and nonhosts—can be included explicitly in epidemiological models,^12^, ^43^, ^44^ though modeling multiple species interactions can become mathematically limiting.^45^

Epidemiological models can be further expanded to incorporate within‐host microbial diversity. The simplest example of this would be coinfection models in which a host is simultaneously infected by two pathogen species.^46^, ^47^ Coinfecting pathogens can increase disease in the host when working synergistically, or lessen disease if pathogens are competing for space or resources, or otherwise working antagonistically.^48^, ^49^ Within‐host models can incorporate multiple trophic levels, considering other interacting microbial species as well the host immune system, which could act as a predator of pathogenic microbes.^49^, ^50^ With the advent of next‐generation sequencing (NGS), information on the composition of host microbiomes is more easily accessible; the challenge is mechanistically understanding how shifts in microbial composition translate into effects on disease development or host health.

### Pathogen influence on host communities: the feedback framework

The feedback modeling framework^22^ was developed in combination with the feedback experimental methodology^51^ to test the potential of the plant microbiome to mediate plant species coexistence. Given this origin, the body of work that emerged from this is often called PSF,^52^, ^53^ but the feedback framework is general, and could also be applied to animal hosts. For plants or animals, feedback occurs when the composition of the host's microbiome changes in a way that alters that host's fitness. Coexistence of two host species depends upon the differential accumulation of microbes on each host species, and the differential responses of the hosts to the differentiated microbes (Fig. 1B). This can be described quantitatively using the interaction coefficient *I*_s_, a summary term representing the difference between heterospecific and conspecific effects of a host and its competitor (Fig. 1B). Specifically, microbiome‐mediated coexistence is possible when there is net negative *I*_s_, or a net negative feedback. This occurs when the negative effects of a host species’ microbiota are greatest for conspecific hosts relative to its heterospecific competitors, either by depressing host growth rate or accelerating the competitor's growth rate (formula for *I*_s_ in Fig. 1B).^22^ This simple condition holds true with extension of the model to local‐scale interactions and dispersal,^54^, ^55^ and generally holds true with addition of negative density dependence,^23^, ^24^ and multiple species.^25^, ^26^

**Figure 2:**
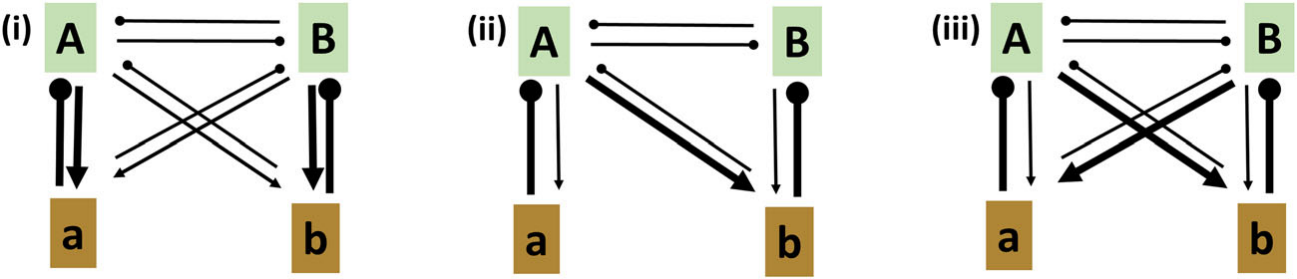
Examples of modules depicting negative and positive feedbacks and how each may emerge due to different tolerances for shared pathogens. Hosts are depicted by capital letters encased in green; pathogens are depicted by lowercase letters encased in brown. The width of the link between organisms depicts the relative strength of the beneficial (arrow) or detrimental (club) effect. Modules represent (i) pathogen‐mediated coexistence via negative feedbacks; the strength of the negative effects on hosts correlates with the strength of positive effects on pathogens; (ii) spillover of pathogen **b** from one host (**B**) to another (**A**), as might occur among phylogenetically related host species. Here, host **A** has high tolerance for (and thus promotes) pathogen **b**, which negatively impacts host **B** to a greater degree than **A**; (iii) asymmetric fitness relationships for two pathogens shared by both hosts in which detrimental effects on neighboring hosts counter negative feedbacks. Such asymmetric relationships can lead to positive feedbacks. In both (ii) and (iii), hosts **A** and **B** will not coexist. Because pathogen **b** in both cases has positive growth rate on host **A**, but a relatively greater negative impact on host **B**, densities of pathogen **b** will suppress host **B**.

Empirically, we find support for a dominant role of pathogens in generating net negative PSF,^56^, ^57^, ^58^ though negative feedbacks can emerge from nonpathogenic components of the host's microbiome as well.^59^ Negative feedback likely results from host‐specific pathogens.^52^ However, positive feedback can result when hosts vary in their tolerance to particular pathogens that spill over onto susceptible competing hosts.^19^ In this case, rather than coexistence, we would see competitive exclusion caused by disease spillover. We can portray the fitness relationship generating these resultant net feedbacks using arrows that depict negative fitness effects within representative feedback modules (Fig. 2).

A major strength of the feedback approach is that the condition for coexistence (*I*_s_ < 0) can be measured through relatively simple experimental approaches: a common microbiome is inoculated onto replicate sterile hosts of two species (plant or animal), allowed to differentiate on these hosts over a period of time, and then the effect of this differentiation on host fitness is assayed in a separate full factorial test evaluating the performance of sterile replicates of each host species when inoculated with the microbiome trained on each host species.^22^, ^51^ Such experiments have provided insights into the importance of pathogens in the maintenance of plant diversity,^19^, ^58^ plant species relative abundance,^57^, ^60^ plant succession,^61^, ^62^ and plant invasion.^58^, ^63^ Consistent with observations that the likelihood of sharing pathogens decreases with plant phylogenetic distance,^64^ the likelihood of net negative PSF increases with plant phylogenetic distance.^58^

### Comparing disease modeling frameworks

The traditional epidemiological and feedback frameworks described above differ in fundamental aspects of their construction that, in turn, define their scope and application. Epidemiological models are population models that aim to quantify disease dynamics; in a community context, they can describe the impact of species diversity and composition on disease incidence. Feedback models, on the other hand, aim to quantify the impact of disease on host species coexistence and host species diversity. While both models represent population dynamics of hosts, they differ in their abstraction of the pathogen dynamics. Epidemiological models focus on disease spread via interactions among hosts as individuals transition between states (e.g., susceptible and infected; Fig. 1A). Unlike epidemiological models, feedback models do not track individual infections or transmission. Instead, the magnitude of disease in the feedback framework is represented as the net effect of pathogens on the average fitness of the host (Fig. 1B).^22^ While all individuals are assumed to experience the average pathogen load and, by extension, similar impacts in the feedback models,^21^, ^22^, ^23^, ^24^ qualitative predictions from these models are confirmed by spatially explicit extensions of the theory, which allow for intrapopulation variation in realized feedback strength.^53^, ^54^

The focus on different host states by epidemiological models allows them to describe the course of an epidemic as it spreads and declines in a population. Consequently, epidemiological models can be used in a predictive capacity to compare how disease could change over time in light of different management strategies, albeit typically only for one or two pathogens at a time.^34^ Feedback models explicitly incorporate multiple direct and indirect interactions (Fig. 1B) that can compensate for or overshadow one another, making it challenging to use for quantitative predictions of the prevalence of any given disease. While there is no analog of *R*_0_ to describe disease spread in feedback models, the interaction coefficient *I*_s_ reflects relative disease impacts on each host, and can be used to predict coexistence between competing host species.

Furthermore, epidemiological and feedback models differ in how they conceptualize transmission. Interestingly, neither model quantifies pathogen abundance per se. And while both frameworks include transmission as a function of encounter between hosts and pathogens, this is explicit in epidemiological models and implicit in feedback models. Certainly, epidemiological models draw attention to many of the biological processes involved in disease transmission among individuals within a population, and incorporate the biology of particular pathogens. Under the feedback framework, a pathogen's presence is presumed based on its impact on plant fitness, but knowing the identity of the disease‐causing organisms is not necessary to understand their net effect on disease in host populations, or predict species coexistence in host communities.

## Dilution effect: aligning epidemiological and feedback frameworks

**Figure 3:**
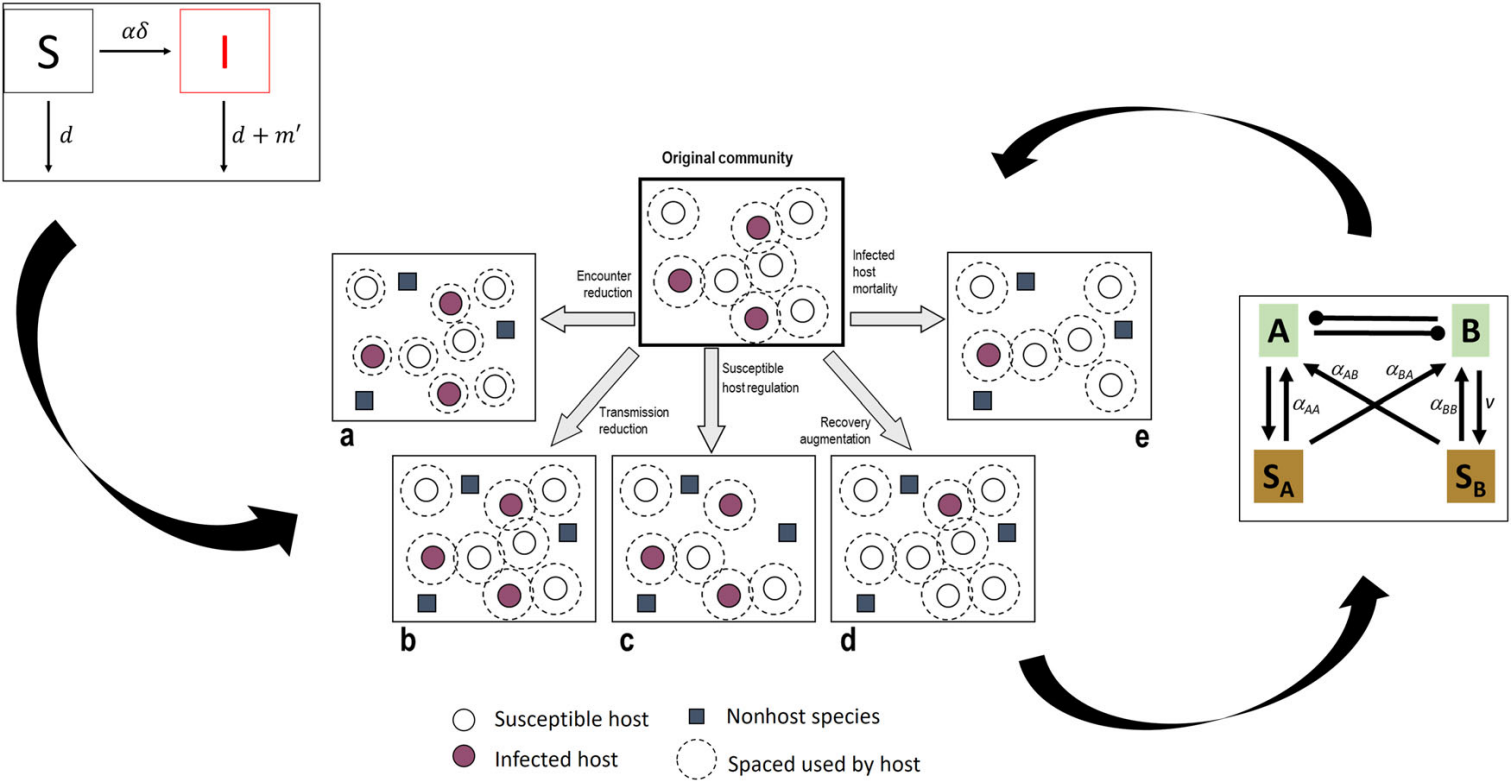
Conceptual diagram of how SI (left) and PSF (right) modeling frameworks interact with potential mechanisms for dilution (center; adapted from Keesing *et al*.^11^). Mechanisms of dilution are grounded in the SI modeling framework (left arrow). Merging dilution and feedback frameworks can both generate new dilution mechanisms (upper right arrow) and inform feedback models (lower right arrow).

Epidemiological and feedback frameworks provide complementary perspectives that together can advance our understanding of disease in natural communities. We demonstrate this using the dilution effect as a case study. Most broadly, the dilution effect reflects the hypothesis that, under some conditions, diverse communities slow disease transmission and that declines in species diversity, therefore, elevate disease risk.^10^, ^11^ Keesing *et al*.^11^ explored potential dilution mechanisms using an SI model of a single, host‐specific pathogen,^11^ from which they inferred the potential effects of species richness and community composition on disease spread. They proposed five key mechanisms by which adding or subtracting nonhost species (macroorganisms) may alter disease risk; each mechanism corresponds to a parameter in their SI model (Fig. 3). Technically, added species can be hosts, just not *amplifying hosts*, but following Keesing *et al*.,^11^ we refer to nonhosts and nonamplifying hosts as “nonhosts” for simplicity. In brief, adding nonhost species to the community can reduce susceptible host availability, alter encounter rates between hosts and pathogens, or modify transmission of the pathogen following encounter. Furthermore, additional nonhost species may reduce disease due to an increase in mortality rates for infected individuals, or an increase in disease recovery rates. Although the term *dilution effect* has been used to describe the general phenomenon of disease reduction with increases in biodiversity, we build on the mechanisms described in Keesing *et al*.,^11^ which are associated with the transmission of a single, host‐specific pathogen.

Examining the mechanisms of dilution in the context of feedbacks makes sense because dilution is most likely to occur in host communities where the transmission of pathogens is a function of host frequency, and where pathogens have host‐specific effects.^37^, ^42^ These conditions are also necessary for pathogens to stabilize host species coexistence via feedbacks.^22^, ^25^ However, while studies of dilution effects often address vector‐borne and other directly transmitted diseases, feedbacks provide the opportunity to examine dilution in systems governed by free‐living pathogens. Furthermore, because feedback models incorporate the complexity of community interactions inherent to dilution mechanisms, they are likely to provide unique insights that increase our ability to predict situations under which we expect dilution to occur.

Keesing *et al*.^11^ proposed dilution mechanisms that focused on changes in species richness that result from adding nonhost species (macroorganisms) to the community. Aligning the parameters from Keesing's original model with the feedback framework requires that we consider the pathogen (microorganism) community explicitly, referred to as intrahost dilution effects in recent empirical studies.^65^, ^66^ To do this, we use representative feedback modules akin to those in Figures 1B and 2. We begin with the simplifying assumption that pathogens are strict host specialists, and then relax this assumption to explore the impact of pathogen specialization on our ability to predict dilution. When possible, we briefly illustrate mechanisms using empirical examples. We focus on PSFs because of the tractability and legacy of PSF research, but we reiterate that the feedback framework applies to both plants and animals.

### Encounter reduction

**Figure 4:**
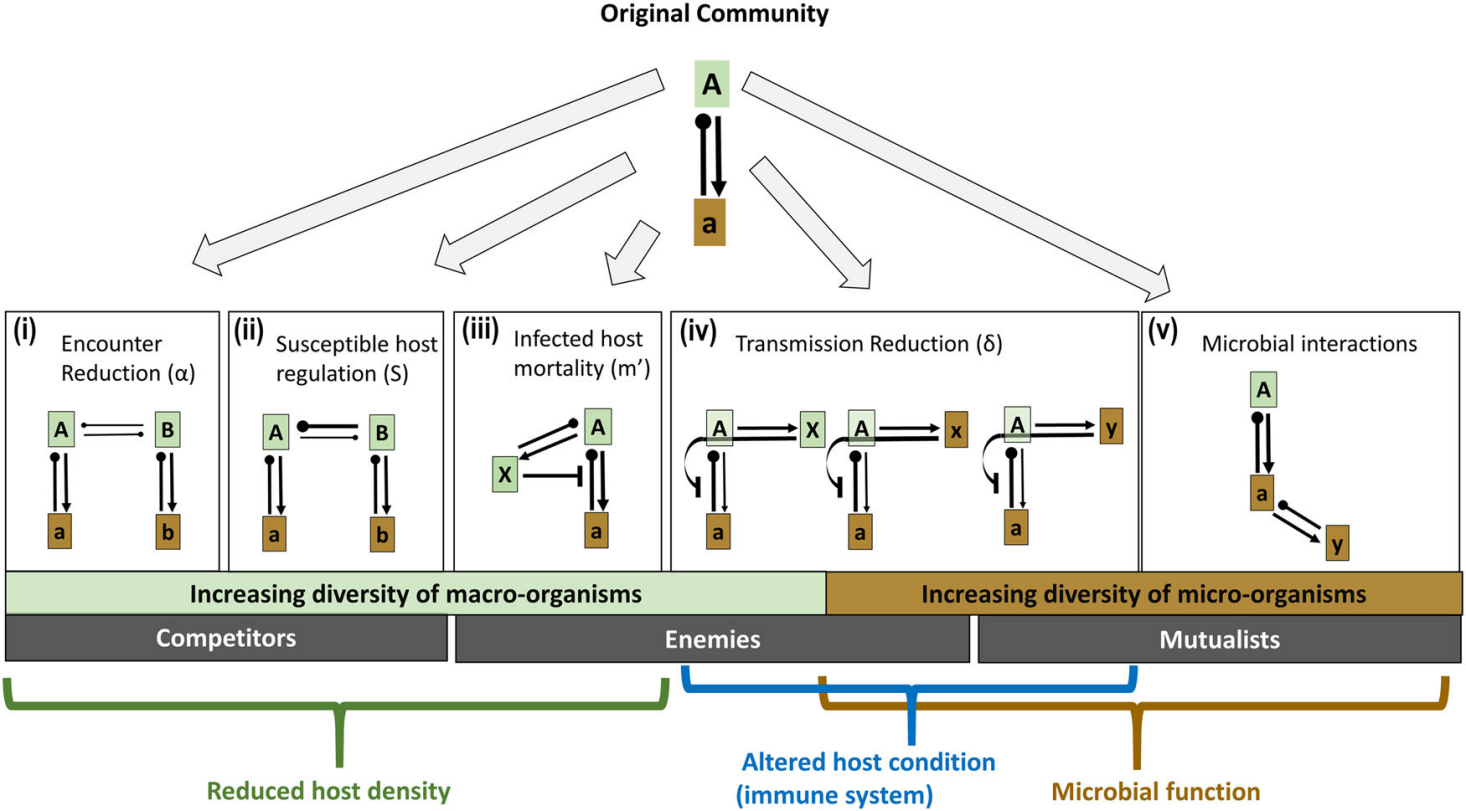
Conceptual model showing mechanisms by which dilution operates in the context of feedbacks. Terms and parameters for dilution mechanisms follow Keesing *et al*.^11^ and Figure 3. Capital letters denote macroorganisms; lowercase letters denote microorganisms. Dilution is considered from the viewpoint of host **A**. The original community contains only one species (**A**), with specialist pathogen (**a**). (i) The addition of a species (**B**) into the host community reduces the density of the host (**A**), which leads to a decline in relative abundance of the specialist pathogen (**a**), and a decline in host–pathogen (**A**–**a**) encounters. (ii) Competitors to the host or (iii) mortality of infected individuals via a predator (**X**) can also reduce host density (green bracket), lowering encounter rates. As pictured here, **B** is a direct competitor for space, or an exploitive competitor for resources, while **X** is a predator that interferes with disease transmission by increasing host mortality of diseased individuals. (iv) Adding species into other trophic levels of the community (predator **X**, pathogen **x**, and mutualist **y**) could reduce disease transmission by modifying host state, for example, induced defenses (depicted by the arrow going through the host), thereby reducing the growth rate of the pathogen on that host. (v) Adding taxa into the microbiome (**y**) increases the chance of direct, antagonistic interactions among microbial taxa that may indirectly benefit the host. Reduced pathogen load on **A** could occur without the addition of a host species, as would be the case if a mycoparasite (coded as **y** to reflect it is an indirect mutualist benefiting **A**) consumes a specialist pathogen, thereby reducing disease. Although in this figure pathogens are strict specialists, this need not be the case for dilution to occur via feedbacks (see the main text; Figs. 1B and 5). Feedback modules in iii–v are adaptations of the basic feedback module presented in Figures 1B and 2 that are designed for heuristic purpose. As such, the modules do not have matching mathematical models or equilibrium solutions. Pairing modules with each of the dilution mechanisms based on epidemiological parameters reveals that, in the context of feedbacks, dilution can occur by adding species to the macro‐ or microorganism communities, which reduces host density, alters host condition, or invokes functions by particular microbes. These nonexclusive pathways to dilution depend on whether added species are competitors, enemies, or mutualists.

Fundamentally, models of negative feedback assume that dilution effects occur via changes in *encounter rate* (Fig. 4i). Feedbacks assume that pathogen abundance increases in proportion to host density and overall total host density is capped (i.e., communities are substitutive), leading to the following dynamic: adding a competing species (i.e., the same trophic level as the host) to the community reduces the relative density of the host at equilibrium. Decreasing density of the host, in turn, reduces the proportion of the microbiome composed of specialist pathogens of that host, as well as their negative effect on host growth. In other words, the dilution effect occurs in negative feedbacks due to a decline in density‐dependent pathogen attacks on the host.^22^ In our example (Fig. 4i), we assumed that competing hosts **A** and **B** are symmetric in their negative effects on one another, and each supports host‐specific pathogens with equivalent negative effects. The presence of **B** reduces the relative density of host **A** from 100% to 50% at equilibrium, with a corresponding reduction in pathogen density and, subsequently, the pathogen's impacts on host **A**’s fitness. In this simple example, **A** and **B** do not share pathogens. However, shared pathogens can still mediate coexistence as long as pathogens differ in their relative effects on different host species (*I*_s_ < 0; Figs. 1B and 2).^22^, ^67^

In PSFs, the addition of heterospecific neighbors leads to less frequent encounters between pathogens and hosts, driven by the heterogeneity in the soil microbiome these new species generate. As with disease in aboveground plant communities,^68^, ^69^ heterospecific neighbors provide barriers that slow or prevent the spread of disease belowground, while cultivating their own host‐specialized communities. Therefore, diverse plant species mixtures also enable plant roots to access soils that contain fewer host‐specialized enemies.^70^ The spatial signatures of negative feedbacks are evident in grasslands^71^ and tropical^57^ and temperate forests,^72^ consistent with predictions of the Janzen–Connell hypothesis.^15^, ^16^ Still, even when the microbial communities of different hosts are well mixed, dilution occurs due to reduced relative abundance of specialized pathogens.^73^, ^74^, ^75^, ^76^, ^77^

### Susceptible host regulation

Adding a species on the same trophic level that is also a stronger competitor can suppress the density of the focal host species, reducing disease pressure for that host (susceptible host regulation; Fig. 4ii). In our example, the magnitude of the arrows between **A** and **B** indicates asymmetric competition, with **B** negatively impacting the fitness of species **A** to a greater degree than **A** impacts **B**. Assuming the relative effect of pathogens on each host species is equal, we would expect species **A** to have a lower density than **B** at equilibrium. However, this change in equilibrium densities may not occur if there are simultaneous changes in host–pathogen fitness relationships.^23^, ^24^

Widespread empirical evidence exists showing that competition between plant species can negatively impacts plant biomass, population growth rate, abundance, and spatial distribution.^78^ Theoretical studies show that the negative effects of competition on host density may be compensated for by benefits of reduced disease pressure (i.e., dilution via susceptible host regulation), thereby allowing an inferior competitor to persist.^23^, ^24^ Competition and PSFs occur simultaneously in nature, often acting additively or synergistically to suppress plant biomass.^79^ However, how commonly PSFs counter competitive inequalities in natural communities (and thus competitor‐driven dilution effects) remains unknown.^79^

### Infected host mortality

In SI models, dilution occurs if added species increase the mortality of infected individuals, leading to reduced disease transmission. For instance, if predators consume diseased individuals preferentially, adding a predator will reduce the density of infected hosts relative to uninfected hosts.^80^ We can represent this within a feedback module by adding a predator (**X** in Fig. 4iii) that interferes with disease transmission, but does not alter the underlying fitness relationship between the host and pathogen (thus the magnitude of the arrows between **A** and **a** does not change). If predation decreases the density of **A**, the realized benefits to its specialized pathogens would also be diminished, leading to lower density of the pathogen. Thus, adding a predator that increases the mortality rate of infected individuals would result in less disease. However, dilution may be transient because decreasing the rates of spread of the disease may feed back positively on the host. Such tritrophic interactions involving organisms outside the basic feedback model (Fig. 1B) make the equilibrium density of **A** and the equilibrium disease level difficult to predict.^81^, ^82^

Predator‐induced mortality is not often considered in PSF studies, perhaps because for adult plants, herbivory typically reduces biomass without causing mortality. Effects of the predator on host defenses, which also reduce disease, are addressed below (see section “Transmission reduction” below). However, in the context of PSFs, dilution via infected host mortality can occur when herbivore attack in combination with disease leads to higher host mortality,^83^ or if herbivores preferentially attack infected plants.^84^ In theory, increased predation on the focal host may follow establishment of another host species that attracts a generalist predator (apparent competition).^17^ Even without an additional host species, the predator reduces disease incidence if predators reduce host density.

### Transmission reduction

Adding a species may reduce the chance of transmission, despite high encounter rates between hosts and pathogens. This is particularly true if the added species is a microbe that modifies the physiological condition of the host. In Figure 4iv, we adapted the feedback module to include an herbivore **X**, a microbial pathogen **x**, or a microbial mutualist **y** whose presence alters the host immune system, reducing the growth rate of the pathogen specialized on that host (thereby enhancing disease resistance). In contrast to adding a predator that interferes with disease transmission by consuming the host (Fig. 4iii), the arrow between **A** and **a** in the case of reduced transmission is narrowed. This reflects that the fitness relationship between the host and pathogen shifts because of the added species acting through the host by altering host physiology. As was the case with the predator in Figure 4iii, the counterintuitive result in the long term may be that with reduced disease in the presence of the microbial mutualist, host density may increase, followed by increases in pathogen abundance. The net realized effect is challenging to predict given the complexity of tritrophic interactions, but the dilution effect via transmission reduction will occur at least transitionally.^85^, ^86^

Given that signaling pathways in plants that are triggered by herbivores, pathogens, and microbial mutualists overlap,^87^, ^88^ dilution via reduced transmission seems likely. However, studies of tritrophic interactions mediated by plant defense have focused more on the effect pathogens have on herbivory than the effect of herbivory on disease,^89^ and even less is known within the context of feedbacks. One exception is a recent study by Bezemer *et al*.,^90^ suggesting that herbivory can alter plant responses to soil biota and the strength of the feedback. They speculate that herbivory alters the composition of pyrrolizidine alkaloids in roots, which can, in turn, alter the fungal community (including pathogens) in the rhizosphere.

Early arriving pathogens can reduce susceptibility to subsequent infections by inducing defenses.^91^, ^92^ Microbial mutualists, such as mycorrhizae, can also protect plants from pathogens,^93^, ^94^, ^95^ as well as influence feedbacks.^23^, ^96^ In feedback models and experiments to date, immunological aspects of feedbacks are typically subsumed in the fitness effects that the microbiome has on its host.^90^ This currently limits our ability to implicate the predator, coinfecting pathogen, or microbial mutualist as the source of a dilution effect.

### Microbial interactions

Adding microbial taxa into the microbiome, regardless of whether or not another host species is added to the community of macroorganisms, can increase direct interactions among microbes in ways that reduce disease (Fig. 4v). For instance, antagonistic interactions among microbes may suppress host‐specialized pathogens. Although species are added to the microbial community in scenarios we describe for both transmission reduction and microbial interactions (Fig. 4iv versus v), we distinguish these mechanisms because in the case of transmission reduction, dilution effects of additional microbial taxa are indirect, mediated by host physiology. This is opposed to dilution caused by direct and antagonistic interactions among microbes.

Microbial interactions are well known to drive disease suppression in agricultural systems^97^ and are, therefore, expected to be important in modifying feedback in natural systems. Using metagenomic tools, microbial taxa are increasingly identified in studies of PSFs.^76^, ^77^, ^98^ Still, it remains challenging to implicate particular taxa as drivers of the negative feedback, much less examine the antagonistic relationship between two pathogens or two microbes more generally. Microbes that inhibit fungal pathogens, such as mycoparasites (e.g., *Trichoderma* species)^99^ or disease‐suppressive bacteria,^100^, ^101^, ^102^ reduce disease in the soil microbiome and could, therefore, influence the magnitude or direction of feedbacks. Detecting the presence of indirect mutualists (from the perspective of the host) may be a first step toward understanding the role of microbially driven dilution effects in the context of PSFs.

### Recovery augmentation

Keesing *et al*.^11^ proposed that increasing recovery rates will slow disease (Fig. 3). For many organisms, complete recovery (total elimination of the pathogen) may never occur. However, recovery can be represented by tolerance of **A** for **a**, due to the introduction of an added intrahost organism, such as a microbial mutualist that reduces the negative effect of pathogens on the host. Increasing host tolerance could allow pathogens to build up to high levels.^85^, ^86^ Similarly, if the added species was a tolerant competitor in the host community (e.g., **B** in Fig. 2ii), this too would amplify disease (see below). In SIR models, increasing the number of recovered hosts lowers overall disease risk, assuming they retain immunity indefinitely; in feedback models, recovery in the form of tolerance will allow the density of hosts to increase, in turn increasing the capacity to support pathogen populations. Consequently, recovery augmentation illustrates the incongruence between conclusions drawn about diversity–disease relationships when considering parameters from feedback and epidemiological models.

### Interactions among mechanisms

Mechanisms for dilution are not mutually exclusive and it is likely that several (or all) happen simultaneously, reducing disease at high plant diversity. For instance, both Mommer *et al*.^76^ and Wang *et al*.^77^ found a decline in the relative abundance of pathogens when plants were grown in polycultures, consistent with dilution via encounter reduction (Fig. 4i). However, microbial composition also changes as host communities become more diverse,^76^, ^103^, ^104^ so it is also possible that interactions among microbes, particularly soil bacteria associated with pathogen suppression^105^ or coinfecting microbes that prime plant defenses,^91^ drive dilution in diverse plant communities (Fig. 4iv and v).

Similarly, mechanisms may operate synergistically. For instance, an early‐arriving pathogen may alter infection by a later‐arriving pathogen (transmission reduction), but also induce a stress response in a plant that triggers an increase in the abundance of disease‐suppressive taxa, reducing disease via direct microbial interactions.^106^ Finally, a single organism may be a diluter via more than one mechanism. For instance, mycorrhizal fungi reduce disease by triggering defense responses in the plant (transmission reduction) as well as competing with pathogens for space or other resources (microbial interactions).^107^ However, it may be difficult, or in some cases, impossible, to tease these mechanisms apart.

### General insights from merging feedbacks with dilution

In most cases, the feedback modules we describe above could be (or have been already) modeled by elaborating on basic epidemiological models. Moreover, in the context of feedbacks, several of the parameters from SI models might collapse into a single dilution mechanism. For instance, for density‐dependent transmission, any ecological process that reduces density of a focal host species will generate a dilution effect (Fig. 4, green bracket). However, pairing feedback modules with each parameter from the SI model highlights different aspects of dilution than the SI‐focused model alone.

Perhaps most generally, the feedback modules make explicit the mechanisms by which increases in diversity due to adding macroorganisms versus microorganisms can generate dilution effects. We can categorize these mechanisms in numerous other ways (Fig. 4, colored bars and brackets). For instance, mapping feedback modules onto the SI parameters allowed us to distinguish dilution mechanisms within the feedback loop, that is, adding species into the same trophic level as the host (Fig. 4i and ii), from dilution mechanisms that result from diversifying the trophic structure outside the feedback loop, such as adding enemies (Fig. 4iii and iv) or microbial mutualists (Fig. 4iv and v). By adding the mechanism “microbial interactions” to the original conceptual model of Keesing *et al*.,^11^ we depart from the epidemiological model because microbial mechanisms do not match a particular parameter in the SI model. However, doing so allows us to further categorize mechanisms according to whether they operate via reduced host density, altering host physiology (i.e., immune responses), or microbiome interactions, including, but not limited to, other pathogens (Fig. 4, colored brackets). Specifically, we can now separate cases in which the effects of adding additional microbial species are mediated by the host via the immune system (transmission reduction; Fig. 4iv) versus direct interactions between microbes, such as competition for resources (microbial interactions; Fig. 4v). These ways of categorizing mechanisms are not mutually exclusive, but increase our ability to formulate predictions about the community composition and environmental conditions under which we might expect to see dilution.

## Dilution via encounter reduction along a gradient of host specificity

Keesing *et al*.^11^ provided a suite of mechanistic explanations for dilution built from single host–pathogen scenarios. However, pathogens differ in their level of host specialization and their relative effects on different hosts. Allowing pathogens to vary along specialist–generalist continuum can advance our understanding of host–pathogen interactions. Furthermore, microbiome studies consistently reveal diverse microbial communities within larger organisms. Feedback theory accommodates multiple pathogens of varying levels of specialization into its predictions for species coexistence. While we illustrated dilution via reduction of host–pathogen encounters using specialist pathogens (Fig. 4i and ii), dilution via encounter reduction can also occur when pathogens are not strict specialists, though in that case, the potential for pathogen spillover onto other hosts necessitates a more precise delineation of exactly what is diluted.

Feedback theory predicts that where pathogens generate negative feedbacks, increasing host diversity will decrease the net effect of pathogens on host fitness.^22^ Based on the single host–pathogen model, Keesing *et al*.^11^ defined dilution as the reduction in disease incidence with increasing diversity. Thus in the single host species SI model upon which dilution mechanisms are based, reduction of disease incidence will also reduce the net effects of the pathogen on host fitness in the population. However, when we add diverse host–pathogen interactions such as those represented in the feedback model, the dilution of disease incidence and the dilution of the net effect of pathogens on hosts may not align.

**Figure 5:**
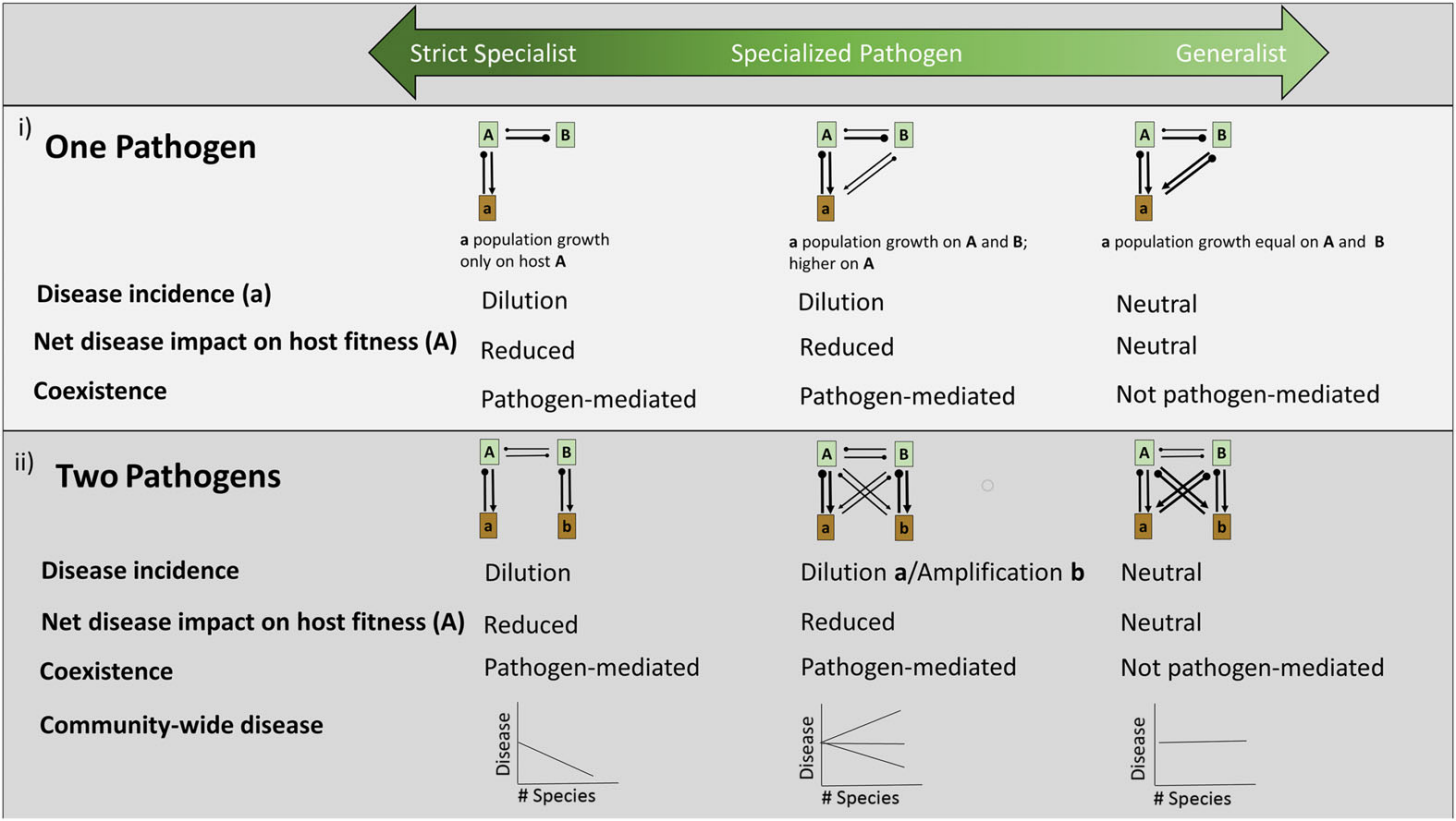
Dilution via encounter reduction, following the addition of a second host species. Disease outcomes are presented for single‐ (i) and two‐pathogen (ii) systems in cases where pathogens are strict specialists (limited to a single host species), specialized (pathogens infect multiple hosts but have different effects on each), and generalists (pathogens grow equally well on each host). Capital letters (**A** and **B**) encased in green signify distinct species in the host community. Lowercase letters encased in brown (**a** and **b**) signify pathogens accumulating on the host of the same letter. The width of the arrows/clubs connecting hosts and pathogens reflects the magnitude of population growth (i.e., fitness benefit or cost, as represented by arrows and clubs, respectively) of the relationship. In these scenarios, we explore consequences for disease (**a**) on host (**A**) when an additional host (**B**) is added to the community. Row titles reflect the blending of epidemiological models (focused on incidence) with feedbacks (focused on coexistence). We distinguish between the impacts of adding a host species on the incidence of a particular disease (caused by a particular pathogen) from the net impacts of disease (which may reflect multiple pathogens) on the host. In the case of multiple pathogens, these impacts may not be the same. Community‐wide disease refers to disease incidence summed across all pathogens.

When considering a single‐pathogen case across a specialist–generalist continuum, both the dilution of disease incidence and dilution of net effects of pathogens on host fitness align (Fig. 5i). In disease systems composed of only one pathogen that is a strict host specialist, adding a nonhost species to the system will cause dilution effects if the density of the focal host is regulated by pathogens (Fig. 5i). If instead of being a strict specialist, the pathogen is *host specialized* such that the pathogen can infect other host species with lower competencies, dilution will still occur, provided that the fitness of the pathogen is greatest on the original host (Fig. 5i). In other words, dilution (and coexistence, in part) occurs because the growth rate of that pathogen differs between hosts. Conversely, if the growth rate of the pathogen is greater on a novel host than the original host, amplification will occur because disease incidence and disease impacts on the original host's fitness would increase. With pathogens that are complete generalists such that added host species are equally competent hosts for the pathogen, adding a new host species will not influence disease incidence because host species are interchangeable. In this case, neither dilution of disease incidence, nor dilution of net pathogen impacts, nor pathogen‐mediated coexistence will occur.

When multiple pathogens infect multiple hosts, the net effects of disease on host fitness may not coincide with patterns of dilution or amplification on disease incidence. As a baseline, consider two pathogens that are strict specialists on separate host species: adding the second host species (**B** in Fig. 5ii) reduces density of the focal host (**A**) and thereby reduces both the disease incidence of **a** and the net effect of disease on that host. In this scenario, both definitions of dilution still apply. Importantly, pathogen **b** will initially be amplified as it increases in abundance following the increase in its host's density, but at equilibrium, negative PSFs operate to dilute each disease (pathogens **a** and **b**) on their respective hosts. In cases where two pathogens are *specialized* such that they are shared between hosts but with differential effects and growth rates on each, **a** will be reduced as the density of **A** declines (dilution). By contrast, pathogen **b**, which is specialized on host **B**, will increase in the community and on **A** (amplification). Collectively, the net effect of disease on the focal host (**A**) is reduced more than when pathogens are host‐specific because total disease pressure includes two pathogens, one of which is less damaging to fitness and yet replaces the other pathogen. So, there will be dilution of the net effect of pathogens on host **A**’s fitness, despite the amplification in incidence of **b**. Here, the two definitions of dilution (dilution of disease incidence versus net pathogen impacts) do not align. In the case where all pathogens are complete generalists, substitution of **A** by another host species will not lead to dilution in disease incidence of either pathogen, will not change the net effect of disease on **A**, and will not lead to pathogen‐mediated coexistence of the two host species.

That species host multiple pathogens—and share pathogens that affect host species differently—helps explain why *community‐wide* disease (i.e., the total amount of disease in a community, across host species) does not always vary across a gradient of host species diversity. In the case of two pathogens that are strict host specialists (Fig. 5i), additional host species may correlate with greater pathogen diversity, but disease incidence and the net effect of each disease on its host may decline due to spatial heterogeneity that generates reduced encounters. When calculating community‐wide disease, we might expect to see a decline in disease incidence due to strict host‐specific pathogens with more host species (Fig. 5ii). Once pathogens are shared, the overall pattern between disease incidence and diversity is more challenging to predict, even in the case of pathogen‐mediated coexistence. For instance, we could see an increase in community‐wide disease with increases in host diversity, if a weak pathogen has a particularly high growth rate on a few individual host species. Alternatively, amplification and dilution of each pathogen incidence on different host species may cancel one another out when summing across all hosts, leading to no overall pattern between community‐wide disease and diversity (Fig. 5ii). Consequently, plant diversity potentially leads to different patterns in disease incidence for a single pathogen than we might see by examining net effects on host fitness or community‐wide disease.

## Complementary versus selection effects in diversity–disease relationships

Biodiversity is presumed to provide numerous ecosystem functions, including primary productivity,^108^ and under some circumstances, enhanced disease reduction.^1^ Given that pathogens can enhance host diversity by mediating species coexistence,^19^, ^58^, ^79^ then reducing pathogen impacts via dilution will likely generate productivity benefits that increase as host species diversity increases. In other words, the benefits of PSFs and dilution effects will increase with host diversity. In both diversity–productivity or diversity–disease relationships, we can assess whether the effect of diversity on the ecosystem service depends on the identity of species added to the community because particular species play a disproportionate role in improving ecosystem function (selection effects) or is a consequence of the number of species per se (complementarity).^42^, ^109^, ^110^, ^111^

Examining dilution through the lens of feedback theory reveals that selection and complementarity in the diversity–disease relationships will likely depend on the trophic level of the species added to the community. Where negative feedbacks operate, adding host species within the same trophic level leads to a dilution effect via encounter reduction, regardless of the identity or function of the new species. In other words, diversity–disease relationships will be due to richness per se when the conditions for host species coexistence via negative feedbacks are met (*I*_s_ < 0), and these diversity effects will be due to complementarity.

By contrast, species added to trophic levels other than the host are likely to generate selection effects in diversity–disease relationships because the direction and magnitude of dilution relies on the identity and function of the species. To reduce disease, an added microbe either needs to protect the host or act antagonistically on the pathogen (Fig. 4iv and v). Consequently, simply increasing the number of microbial taxa alone will not necessarily lead to dilution. However, the probability of the microbial community containing a protective or suppressive taxon will increase as the number of microbial taxa increases. The same principle holds true for macroorganisms outside the trophic level of the host.

Disease plays a key role in the positive diversity–productivity relationships as well. Studies that manipulate pathogens have found that soil pathogens suppress productivity at low plant diversity, and that the effects of pathogens are ameliorated at high levels of plant diversity.^73^, ^112^ Studies of diversity–productivity relationships that included feedback experiments^74^, ^75^, ^113^ confirm that specialized pathogens operating through negative PSFs drive this pattern. Feedbacks between hosts and the soil microbiome cause transgressive overyielding, indicating positive complementarity.^77^, ^114^ Pathogen‐mediated complementarity in diversity–productivity relationships provides evidence of pathogen dilution, and evidence that dilution is a causal mechanism for the productivity benefits that accrue at higher host diversity.

## Predictions generated by integrating epidemiology and feedbacks

Although dilution effects have typically been described as a consequence of increasing biodiversity,^11^ viewing dilution through the lens of feedback theory provides a framework for exploring ways in which dilution also maintains local diversity. This is possible because feedback theory incorporates dynamics of both the host and microbial communities, as well as the indirect effects that can influence community‐level outcomes. Most fundamentally, aligning the feedback and SI‐grounded dilution frameworks reveals that the conditions for pathogen‐mediated species coexistence are also conditions under which we can expect dilution of the net effect of pathogens on their hosts.

A major strength of the feedback framework for exploring disease in natural communities is its connection to empirical tests using PSF experiments. Widespread, compelling evidence has accumulated that PSFs are negative and can facilitate species coexistence^22^, ^58^ and structure plant communities.^51^, ^57^, ^60^, ^115^ We can, therefore, rely on empirical studies of PSFs for evidence of dilution, as well as to generate and test new predictions for when dilution should occur in plant communities. Interpreting empirical evidence of PSFs in the context of dilution leads us to five predictions regarding general patterns in the relationship between disease and diversity.

### Dilution effects will be greatest in the most productive environments

Dilution effects are likely where pathogen‐generated negative feedbacks mediate host species coexistence. Recent results suggest that pathogen‐mediated coexistence is more likely in warmer and wetter environments: conspecific negative density dependence in forests is generally greatest in the southeast of the continental United States^72^ and in tropical latitudes.^116^ Theory indicates that this conspecific negative density dependence is sufficiently strong to explain the increasing plant diversity along these environmental gradients,^25^ and pathogens are thought to be the major driver of this conspecific negative density dependence.^19^, ^57^ Given this evidence, we suggest that pathogen dilution effects are likely to be strongest among native plant species in the most productive environments.

### Dilution and addition of phylogenetically divergent species

Whether dilution occurs depends in part on whether adding a species to the host community increases or decreases *community competence*, that is, the fraction of the community that supports population growth of a particular pathogen (Table 1). Dilution will be strongest when adding species decreases community competence, because this will lead to greater dilution of incidence via encounter reduction. Soil‐borne pathogens that specialize on a single plant species, or even a genus, are rare^117^; consequently, not all species added to the community will increase community competence to the same degree. In plant communities, community competence partly reflects phylogenetic diversity among hosts, because host species that are evolutionarily closely related are more likely to share pathogens.^64^, ^118^, ^119^ Indeed, the phylogenetic diversity of host communities predicts community‐wide disease for foliar pathogens,^120^ and initial host diversity reduced the community‐wide parasite load (insects and pathogens) in plant communities after a period of 2 years.^121^ Empirical studies of feedbacks show that plants grow less successfully in soils conditioned by closely related species^122^ and stronger negative feedbacks occur between distantly related plant species.^58^ Collectively, these observations suggest that dilution will be strongest with addition of a host species that is phylogenetically divergent from resident hosts, as supported by the recent work in animal systems.^123^

**Table 1:** Glossary of key disease ecology terms used in this paper

Term	Definition
Amplifying host	Infected host with a high transmission rate.
Coinfection	Simultaneous infection by more than one pathogen. Coinfection is easily accommodated in PSFs, as net effects of microbiome differentiation on host species are represented.
Community competence	Community's capacity to support disease; sum of each hosts species’ competence multiplied by that species’ abundance.
Competence	Ability of a host to support a pathogen and effectively transmit infection to another susceptible host or vector.
Community‐wide disease	Sum of disease incidence across all species and all diseases.
Density‐dependent transmission	When the amount of disease in a population depends on the number of infected individuals. PSF theory assumes density‐dependent transmission.
Disease incidence	Number of new disease cases in a population in a given time period. In PSFs, disease impact is assumed to mirror host density. Infection in individual plants is not tracked.
Disease prevalence	Proportion of infected individuals in a population. In PSFs, prevalence is not directly measured; rather, net impact of disease on host fitness is.
Environmental transmission	Transmission of disease via environmental sources (such as soil or water) rather than through direct host contact or a vector.
Frequency‐dependent transmission	When the amount of disease in a population depends on the proportion of infected individuals. While PSFs arise from density‐dependent transmission, negative feedback generates negative frequency dependence when communities are assumed to be substitutive.^22^, ^150^
Host‐specific pathogen	Pathogen that only attacks a single species.
Host‐specialized pathogen	Pathogen that can theoretically attack multiple species but has a differential impact on these species.
Infected host	A host with a pathogenic organism living on or in it. PSF theory assumes that all infected hosts are competent hosts, and can transmit diseases.
Interaction coefficient (*I*_s_)	The metric derived from PSF theory that contrasts conspecific and heterospecific impacts of microbiome on coexistence.^22^
Negative feedback	The density‐dependent accumulation of pathogens on hosts will limit the growth rate of that host as it reaches high density. With such negative feedback (*I*_s_ < 0), pathogens can mediate coexistence of host species.
Pathogen	An organism that causes disease while living in or on a host.
*R*_0_	Number of infections caused by the introduction of one infected individual into a wholly susceptible population.
Recovery	Elimination of disease from an infected individual. PSF theory does not explicitly consider host recovery, though disease impacts are assumed to decline with host density.
Resistance	The capacity of a host to reduce the extent of pathogen infection by preventing infection or limiting pathogen growth.
PSF	The feedback on plant fitness due to changes in the soil community composition.
Spillover	Transmission of an abundant pathogen from one host population into a novel host population. PSF theory integrates the net effect within disease effects on conspecific as well as on heterospecific hosts, thereby accounting for spillover effects on host species coexistence.
Susceptible host	Species (or individual) that can be infected by a pathogen. In a PSF framework, susceptibility is typically considered on a species, rather than on an individual level.
Tolerance	Ability of a susceptible host to allow some level of pathogen infection with minimal impacts on host fitness. In PSFs, tolerance to a pathogen is the closest analog to recovery.
Transmission	Movement of pathogens between hosts. While transmission is explicitly modeled in epidemiological models, net impacts of disease are assumed to be density dependent in PSFs.

note: Along with definitions, brief discussions of differences in term usage between epidemiological and feedback models are provided when applicable. PSF, plant–soil feedback.

### Nonnative plant species, dilution effects, and competence for resident pathogens

Whether or not a nonnative plant species generates dilution effects depends on its phylogenetic relatedness to the existing plant community, as well as its defense traits. Together, these factors determine the competence of a nonnative species to host the existing suite of soil pathogens that drive feedbacks.

If the nonnative species is distantly related evolutionarily, increasing diversity in a community by adding a nonnative species will dilute disease incidence for resident native host species by reducing pathogen encounter. The native and nonnative species will coexist via negative feedbacks if the nonnative also suffers negative density dependence (via pathogens or otherwise). If the nonnative species experiences enemy release,^124^ it may induce dilution for the resident host species, but the resident native species may not persist in the face of competitive pressure. If an invading non‐native species is closely related to resident species, it may be more susceptible to their pathogens and amplify disease for the native host (spillback).^125^

Pathogen‐mediated coexistence occurs widely in plant communities dominated by native species, but appears less influential in communities composed of nonnative species.^126^ It follows that dilution effects will be most profound where native host species–pathogen interactions dominate in the resident community.^58^

### Adding species to host trophic level, complementarity, and dilution effects

Few studies have partitioned diversity–disease relationships according to selection and complementarity effects,^42^ but compelling evidence is emerging that the degree to which complementarity contributes to dilution effects varies depending on whether the added species is of the same trophic level as the resident host.

For instance, Becker *et al*.^111^ found that increasing the host diversity of amphibian hosts slowed the spread of *Batrachochytrium dendrobatidis*, primarily due to complementary effects. In this case, adding more host species altered habitat use, minimizing the contact between hosts due to partitioning habitat. When Johnson and Hoverman^65^ manipulated the diversity and composition of parasites in a single amphibian host species, individual parasite infections and total disease incidence declined as parasite diversity increased. However, host fitness was reduced when exposed to the most diverse parasite communities because more diverse parasite assemblages had a greater chance of containing the most virulent parasite species, that is, the selection effect. Furthermore, if adding species to the same trophic level as hosts generates dilution via complementarity, we might further expect that adding a species into the same trophic level as the host will generate dilution effects more reliably than adding species into a different trophic level.

### Global changes, negative feedbacks, and diversity–disease relationships

Disease requires not only a susceptible host and virulent pathogen but also an environment conductive to disease development (disease triangle).^127^ Thus, we expect global changes to alter disease dynamics, including dilution^120^, ^128^, ^129^ and negative feedbacks.^130^ However, specific predictions are challenging because all trophic levels, and thus all dilution mechanisms (Fig. 4), are potentially affected. Most fundamentally, we can expect that global changes that disrupt negative feedbacks will also reduce dilution effects.

For instance, global changes that restrict host diversity will limit dilution via encounter reduction. For negative feedbacks to operate, heterospecific hosts must be available to colonize a site. Fragmentation alters species richness and composition^2^ in part by disrupting dispersal,^131^ which could limit dilution via encounter rate reduction. If land use degradation limits phylogenetic diversity regionally^132^ or favors poorly defended, early‐successional species adapted to disturbance,^61^, ^133^ the likelihood of a phylogenetically distinct species arriving to a site will decline.

If global changes alter microbial diversity and composition, even without reducing host diversity, the diversity–disease relationship will likely be affected. The composition of pathogen communities reflects drift, selection, and dispersal in addition to interactions with host defenses.^134^, ^135^, ^136^ Furthermore, the sequence and timing of microbial species’ arrival influences community structure and disease dynamics in soil and plant microbiomes.^137^, ^138^, ^139^, ^140^, ^141^ Thus, anthropogenic activities that alter relative abundance of microbial taxa or the likelihood for successful dispersal would theoretically impact both negative feedbacks and dilution effects. Although fragmentation can limit the diversity and composition of some beneficial microbes^142^, ^143^ and weaken plant–enemy interactions,^144^ we have much to learn about the cumulative impacts of anthropogenic changes on soil microbiota and their subsequent impacts on PSFs.

Finally, increased climate extremes and variability could also disrupt native host–pathogen interactions in ways that change the direction or magnitude of diversity–disease relationships. Precipitation and temperature influence fungal composition,^145^, ^146^ fungal activity,^147^ and extreme conditions, such as drought generate shifts in microbial communities that alter PSFs.^148^ Furthermore, climate variability—not just abiotic extremes—shifts the direction of feedbacks from negative to neutral or positive,^149^ thereby reducing the dilution effects.

## Conclusion/synthesis

Keesing *et al*.^11^ proposed a suite of mechanisms for dilution effects via parameters in SI models, exploring the numerous ways in which richness and composition influence disease incidence. The feedback framework was originally developed to identify conditions for pathogen‐mediated coexistence among host species. The conditions for pathogen‐mediated coexistence via negative feedbacks are also conditions that generate dilution effects in the net effect of disease on host fitness via encounter reduction. Intersections between these two frameworks reveal several key insights. First, when additional species are added to the host communities in which negative feedbacks are operating, dilution effects are likely. When the number of species increases due to additional species in other trophic levels, dilution effects may be transient, and long‐term outcomes are challenging to predict. Finally, when multiple diseases and multiple host species are present, whether dilution occurs depends on whether the focus is on individual pathogen incidence, the net effect of disease on host species, or community‐wide disease incidence. Because the net impact on host fitness can be diluted, even while particular pathogens are amplified in the system, quantifying dilution for a single host species by grouping diseases (e.g., when identifying disease by a symptom common to more than one disease) overlooks the aspects of dilution that focus on transmission dynamics— that is, why disease prevalence may vary with host species diversity. Moreover, when pathogens are shared among species, amplification and dilution of particular pathogens can cancel one another out such that we see no pattern in community‐level pathogen load with increasing diversity. By adding an additional mechanism (microbial interactions) to dilution effects, we highlight the growing necessity of including not just pathogen diversity but also other direct microbial interactions in the microbiome. While we have described ways in which dilution mechanisms may operate in the context of feedbacks (Fig. 4), and highlighted potential outcomes for dilution of disease incidence, net disease impacts on hosts, and community‐wide disease (Fig. 5), the challenge of identifying the conditions under which we may see particular dilution mechanisms and disease outcomes remains.

## Author contributions

C.D.C. and J.D.B. conceptualized the paper and developed the figures, with input from M.H.H. All authors contributed to writing the paper.

## Competing interests

The authors declare no competing interests.
